# Systematic Exploration of Privileged Warheads for Covalent Kinase Drug Discovery

**DOI:** 10.3390/ph15111322

**Published:** 2022-10-26

**Authors:** Zheng Zhao, Philip E. Bourne

**Affiliations:** School of Data Science and Department of Biomedical Engineering, University of Virginia, Charlottesville, VA 22904, USA

**Keywords:** kinase inhibitor, covalent kinase inhibitor, privileged warhead, nucleophile, rational drug discovery

## Abstract

Kinase-targeted drug discovery for cancer therapy has advanced significantly in the last three decades. Currently, diverse kinase inhibitors or degraders have been reported, such as allosteric inhibitors, covalent inhibitors, macrocyclic inhibitors, and PROTAC degraders. Out of these, covalent kinase inhibitors (CKIs) have been attracting attention due to their enhanced selectivity and exceptionally strong affinity. Eight covalent kinase drugs have been FDA-approved thus far. Here, we review current developments in CKIs. We explore the characteristics of the CKIs: the features of nucleophilic amino acids and the preferences of electrophilic warheads. We provide systematic insights into privileged warheads for repurposing to other kinase targets. Finally, we discuss trends in CKI development across the whole proteome.

## 1. Introduction

Protein kinases catalyze the transfer of the γ-phosphate group of ATP to specific substrates, such as serine, threonine, or tyrosine, on target proteins and thus play a vital role in almost all aspects of cell function [1,2]. Abnormal regulation or genetic mutation of protein kinases causes many different diseases, including cancers [3,4]. Therefore, protein kinases have been recognized as important drug targets [5,6]. As such, over the last three decades, tremendous progress has been made in kinase-targeted drug design and cancer therapy [5,7,8,9]. So far, 71 small molecule kinase-targeted drugs have been approved by the Food and Drug Administration (FDA), and more than 300 protein kinase inhibitors have undergone clinical trials since the first small molecule kinase-targeted drug, Imatinib, was approved in 2001 [10,11]. These achievements show that kinase-targeted drug discovery has become a mature field [7,12]. Nevertheless, due to acquired drug resistance and off-target-induced toxicity, more kinase-targeted drugs with desirable kinome-scale selectivity can be expected [6,13,14,15].

With increased numbers and hence understanding of kinase structures, diverse kinase inhibitors, or degraders, have been rationally developed with the desired selectivity and efficacy. These are classified as Type-I/II inhibitors, Type-III/IV (allosteric inhibitors), and PROTAC degraders [5,7,8,16]. Type-I inhibitors typically occupy the ATP binding site. Type-II inhibitors not only bind to the ATP binding site but also extend into the nearby allosteric pockets. The allosteric pocket adjacent to the ATP binding site is the binding cavity for Type-III allosteric inhibitors. In contrast, Type-IV allosteric inhibitors bind to the allosteric pockets located on the kinase surface and far from the ATP binding site. For example, GNF-2 and GNF-5 are two Type-IV allosteric inhibitors binding within the C-lobe [17,18]. Recently, proteolysis targeting chimeras (PROTACs) have been applied to kinase drug discovery as a new treatment modality [16]. Typically, PROTAC degraders are composed of three parts: a protein target binder, a linker, and an E3 ubiquitin ligase ligand. Thus, PROTAC-based kinase degraders not only bind to the kinase catalytic domain using the protein target binder but also bind to the E3 ubiquitin ligase by using the E3 ubiquitin ligase ligand [19,20].

**Figure 1:**
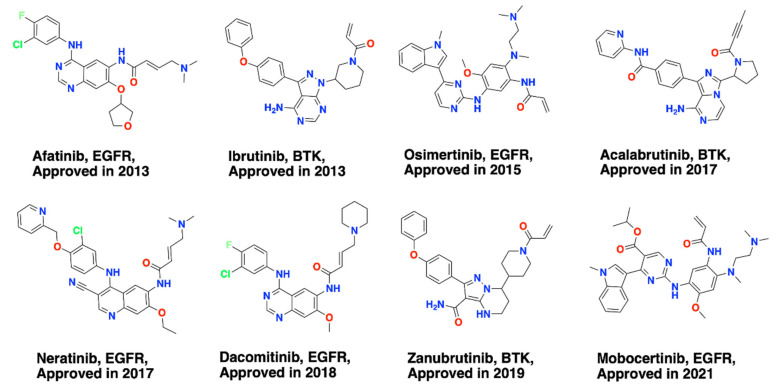
FDA-approved covalent kinase inhibitors with year approved and primary target.

**Figure 2:**
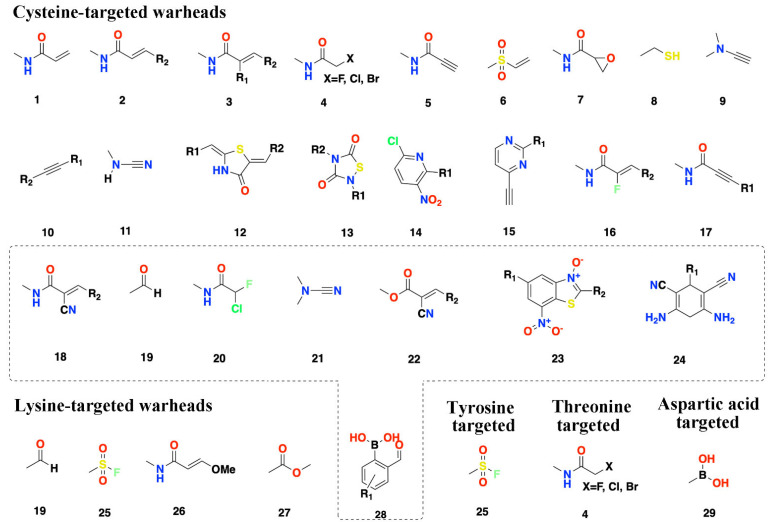
Warheads present in available covalent kinase inhibitors. Warheads are grouped based on different targeted amino acids. The dashed rectangle shows the warheads found in reversible CKIs.

In this paper, we focus on covalent kinase inhibitors (CKIs), which have been attracting attention due to their enhanced selectivity and exceptionally strong affinity [21,22,23,24,25,26,27,28,29]. Typically, CKIs comprise a scaffold and an electrophilic group (also termed “warhead”) to form covalent interactions with a reactive amino acid side chain (nucleophilic group). Eight CKIs have been approved by the FDA (Figure 1), and more than 30 CKIs are in clinical trials [11,30]. Different amino acid side chains, such as lysine, cysteine, aspartic acid, and tyrosine, are used as nucleophilic groups. Different warheads have been developed for use as specific CKIs (Figure 2). Collectively these developments provide valuable knowledge for further CKI development. 

Here we first collate all the CKIs, then we review all CKIs based on their binding positions, binding modes, and binding characteristics. Additionally, we systematically analyze the warhead types and their privileged properties. Finally, we discuss the challenges and design strategies for rational covalent drug discovery.

## 2. Kinetic Mechanisms of CKIs

CKIs are usually small-molecule kinase inhibitors that not only bind to the kinase binding site but also form a covalent interaction with kinase amino acids through a two-step binding process:


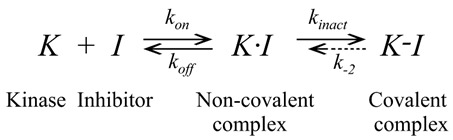


In the first step, the covalent inhibitor binds to the binding pockets through non-bonded interactions, bringing the electrophilic group suitably close to the designated nucleophilic group. The non-covalent complex is formed with a dissociation equilibrium constant $Ki=k_{off}/k_{on}$. In the second step, the non-covalent complex undergoes covalent binding. $k_{inact}$ is the rate constant for the covalent reaction step. $k_{-2}$ represents the reversibility of the covalent reaction [24].

## 3. Current CKIs

We collected all CKIs from recent scientific reviews [5,7,10,23,24,30] from PubMed (https://pubmed.ncbi.nlm.nih.gov, accessed on 20 May 2022) and published databases including CovalentInDB [31] and ACS publications (https://www.acs.org, accessed on 5 June 2022). First, the ACS publications were searched using the query words “covalent or irreversible” and “kinase” and “inhibitors.” The scientific reviews and the database CovalentInDB were manually checked. Since our focus was to explore privileged warheads, only one representative CKI was selected if there were a series of CKIs with the same warheads and reacting with the same amino acids to determine the structure-activity relationship (SAR). A total of 200 CKIs with detailed structural information, such as the chemical structure (SMILES format), warhead type, nucleophilic partner residue, and the positions of nucleophilic partner residues, constitute our database for further analyses (Table S1). 

### 3.1. Kinases and Nucleophiles

**Figure 3:**
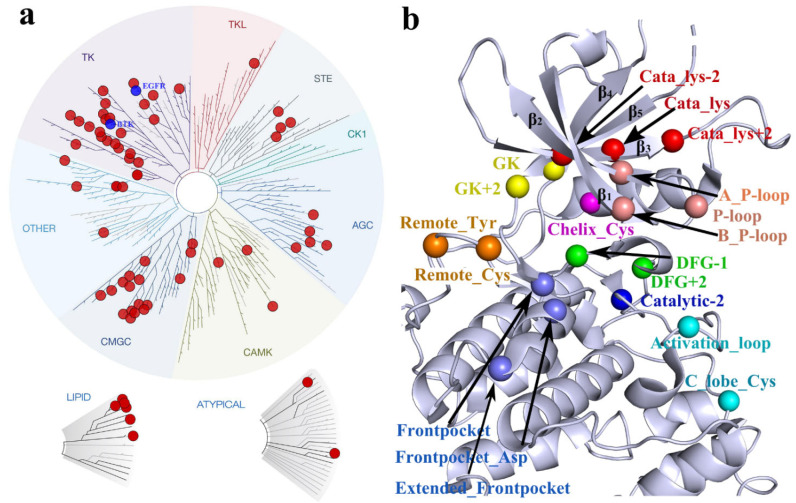
(**a**) Distribution of kinases with at least one CKI (the TREEspot tool www.discoverx.com, accessed on 20 May 2022). (**b**) Distribution of active amino acids as nucleophiles (PDB 5efq [35] as the kinase template).

To date, published CKIs target 69 kinase families distributed across all human kinome groups (TK, TKL, STE, AGC, CAMK, CMGC, other, lipid, and atypical), but excluding CK1 (Figure 3a). Of the 69 kinases, 26 belong to the TK kinase group, including the kinases BTK and EGFR (which have been targeted with 8 FDA-approved covalent kinase drugs (Figure 1), one in the atypical kinase group, and six in the lipid kinase group. Previous studies have demonstrated that there are abundant cysteines available as nucleophilic groups located within different parts of the binding site for about 200 different kinases over the whole kinome [22,32,33]. Thus about one-third (69/200) targeted by CKIs have been identified, implying that although great progress has been made, further efforts to develop CKIs are warranted [12].

We analyzed the positions of active amino acids within the binding sites of different kinases. The secondary structure of the kinase domain is where ATP binds is conserved [34]; however, the distributions of active amino acids are different among different families [32]. We classified all kinase structures that CKIs target based on the positions of active amino acids in the binding sites. There are 19 positions containing active amino acids (Figure 3b). Three positions with such cysteines are in the P-loop (B_P-loop, P-loop, and A_P-loop); three positions on the β3 sheet (Cata_lys-2, Cata_lys, and Cata_lys+2); one position at the end of C-helix (Chelix_Cys); two positions at the hinge (GK and GK+2); three positions at the front pocket (Frontpocket, Frontpocket_Asp, and Extended_Frontpocket); two positions at the DFG peptide (DFG-1 and DGF+2); one position in the catalytic loop (Catalytic-2); one position in the activation loop (Activation_loop); one position located at the C-lobe (C_lobe-Cys); and two positions (Remote_Cys and Remote_Tyr) located at the C-terminus but close to the ATP binding site. In addition to cysteine, which is used as the active nucleophilic group, threonine, lysine, tyrosine, and aspartic acid are also included: tyrosine at the position of Remote_Tyr; lysine at the position of Cata_lys; and aspartic acid at the position of Frontpocket_Asp. There is one position (Frontpocket) where the cysteine is targeted by CKIs, eight of which were approved by the FDA (Figure 1), suggesting this front-pocket position can be successfully targeted by CKIs.

### 3.2. Warheads

The warheads (electrophiles) in CKIs are vital functional groups not only for pairing with active amino acids through covalent bonding but also for determining the reversibility of covalent reactions. There are many viable warheads that have been added to CKIs. Here we summarize 29 types of warheads that have been used in covalent kinase inhibitor design (Figure 2) and also provide information on every CKI (Table S1) for covalently targeting the designated active amino acids. Specifically, warheads **1**–**24** as the electrophilic group binds to the thiol group of the cysteine side chains, warheads **19, 25**–**28** bind to the ε-amino group of the lysine side chains, warhead **4** binds to the hydroxyl group of the threonine side chain, warhead **25** binds to the hydroxyphenyl group of the tyrosine side chains, and warhead **29** targets the α-carboxylic acid group of aspartic-acid side chains. However, warhead **4** binding to threonine and warhead **29** binding to aspartic acid have not been validated by experiments [36,37].

While significant progress has been made in the design and development of covalent kinase inhibitors, covalent-inhibition-induced potential toxicities still exist [21,38,39]. Consequently, reversible-covalent inhibition has received increasing attention [40,41]. Reversible CKIs, as the name suggests, not only form covalent interactions with the kinase but also avoid irreversible kinase covalent modification [41]. Reversible CKIs that have been developed which target at least 10 different kinases [40] using 8 viable warheads (Figure 2) [40]. 

### 3.3. Privileged Warheads

**Figure 4:**
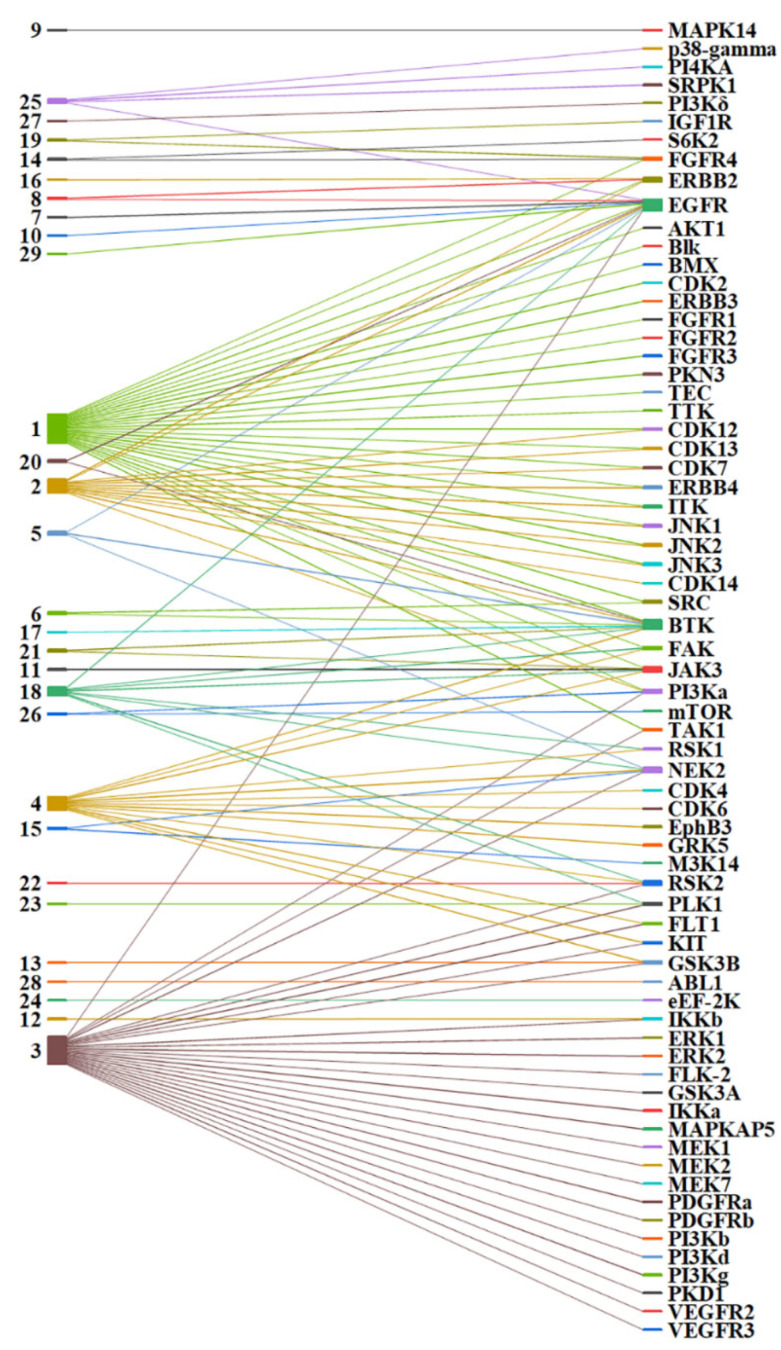
Warheads applied to different kinases.

**Figure 5:**
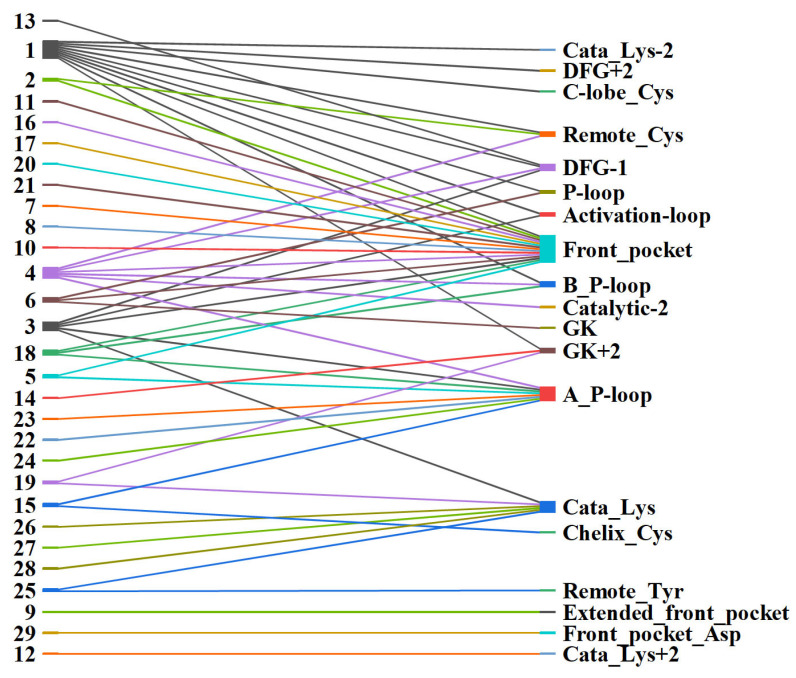
Warheads and their kinase binding sites.

The recent approvals of covalent kinase drugs have prompted additional efforts to design CKIs. Typically, CKIs are rationally designed from a bioactive reversible kinase ligand appended to a warhead that reacts with the proximal amino acid. Strategies for designing warheads are mostly a “trial-and-error” process through determining the SAR of different warheads and identifying the optimal one. This process is expensive and time-consuming. However, efforts to date have provided extensive experimental data that can be leveraged. For example, some warheads show privileged properties, which means the warheads can be used to target more than one kinase and nucleophilic group in different parts of the binding site [42]. Here, we summarize current CKI data and highlight how warheads can be applied to prospective CKI design against kinases known and unknown. It should be noted that in the current data, warhead **29** (boronic acid), binding to EGFR front-pocket Asp800, and warhead **4** (chloroacetamide), binding to CDK4/6 front-pocket Thr102/Thr107, were validated by docking simulations rather than confirmed experimentally [36,37]. Out of 29 types of warheads, 10 warheads (**1**, **2**, **3**, **4**, **5**, **6**, **15, 18**, **19**, and **25**) are privileged as they target more than one kinase and also multiple amino acid positions within the kinase binding sites (Figure 4 and Figure 5). For example, warhead **1** (acrylamide) has been used to target 29 kinases, as shown in Figure 4. 

Out of the 29 kinases, warhead **1,** as an electrophile, forms a covalent interaction with a proximal amino acid cysteine within the corresponding binding site. The formation of covalent interaction follows the typical kinetic mechanisms of CKIs, as forementioned with a Michael addition. Moreover, warhead **1** could also be applied to target the cysteines distributed in 10 different parts of the binding site (Figure 5). These cysteine positions are located at the part of the front pocket (Front_pocket), but also located at the Hinge (GK+2), the P-loop (P_loop and B_P-loop), the activation loop (Activation_loop), the Catalytic loop (Cata_Lys-2), the DFG peptide (DFG-1 and DGF+2), and the C-lobe (Remote_Cys and C-lobe_Cys). Different cysteine positions lead to different intrinsic reactivity due to the local protein environment within the binding site. For example, the front-pocket cysteines are located in an open, solvent-accessible area, but the DFG-peptide cysteines are located at the center of the ATP-binding site, a hydrophobic subpocket [23,43]. Here, warhead **1** targets cysteines at different locations highlighting its privileged properties. For example, covalent binding with the hinge (GK+2) cysteine is very challenging because this cysteine is located above the inhibitors’ hinge-binding motif, which is a highly conserved feature among protein kinase inhibitors forming 1–3 stable hydrogen bonds to the hinge region [34]. The GK+2 cysteine has been successfully addressed, amongst other kinases (TTK and S6K2) [44,45] in FGFR4, which is the only member of the FGFR family of receptor tyrosine kinases having a cysteine at this position. Thus, with warhead **1**, covalent FGFR4 inhibitors have been developed that achieve excellent selectivity over the other family members, FGFR1–3. In contrast, covalent pan-FGFR inhibitors have been developed by targeting a P-loop cysteine common to all FGFR kinases [46,47]. 

Warheads **2**, **3**, and **18** also show privileged properties, targeting more than one kinase and different parts of the binding pockets. Warheads **2**, **3**, and **18** are derivatives of warhead **1**. In warhead **2**, one β-hydrogen at the β-carbon position is substituted by one non-hydrogen functional group, such as methyl. By contrast, in warheads **3** and **18**, two hydrogens at α and β-unsaturated carbons are substituted by non-hydrogen functional groups, respectively. It is worth noting that the electron-withdrawing properties of the cyano functional group promote its reversibility for warhead **18**. Currently, warhead **18** has been applied in designing various reversible CKIs, such as targeting BTK, EGFR, JAK3, and RSK2 [40,41,48,49,50]. One of the reversible CKIs with warhead **18**, Rilzabrutinib (PRN1008) from Principia Biopharma, is a BTK CKI currently in Phase-III trials to treat pemphigus vulgaris and immune thrombocytopenia [51]. 

Warhead **4** is a type of halo-acetamide with an α-halogen substituent, which was used to develop CKIs targeting 13 kinases (Figure 4). Warhead **4** also targets different cysteine positions, such as Remote_Cys, DFG-1, Front_pocket, B_P-loop, Catalytic-2, and A_P-loop (Figure 5). Warhead **5** is a propynamide group targeting the kinases EGFR, BTK, and NEK2, and different cysteines distributed at the A-loop position and the Front_pocket position. The A-loop and the Front_pocket are located on the edge of the binding site and reachable by solvent, which suggests that warhead **5** can form covalent interactions within a polar vibe. Warhead **6** is a sulfonamide moiety that has been used to covalently link cysteines located in the GK and P-loop moieties of the SRC kinase and the Front_pocket moiety of the BTK kinase (See Supplementary Material Table S1). Warhead **15** is a larger ethynylpyrimidine group. Warhead **15** was used to link to the cysteine located at the front pocket, an open, solvent-reached area [52], but also linked to the cysteine located in the deep hydrophobic pocket at the back of the kinase binding site [53]. Warhead **19**, an aldehyde, is a powerful electrophile that supports covalent linkage not only to cysteine but also to lysine. Warhead **19** is often used to design proteolytic enzyme inhibitors [54]. However, it is worth mentioning that aldehyde is not frequently applied in drug discovery because of the unexpected toxic adduct from its additional reactions with other off-targeted enzymes [55]. In CKIs, the aldehyde is used as the warhead to improve the binding affinity and selectivity [56]. Warhead **25**, a sulfonyl fluoride, targets tyrosine at Remote_Tyr and lysine at Catalytic_lys. The lysine located at the Catalytic_lys position is a conserved catalytic residue within the kinase binding site. Tyrosine and lysine provide more nucleophilic resources within the kinase binding sites for designing CKIs beyond the cysteine group which is the most frequent nucleophilic group (Figure 2). In summary, these 10 warheads have proven robust and lend themselves to further CKI development.

## 4. Discussion and Outlook

Covalent kinase inhibitors (CKIs) have proven valuable and viable for treating non-small cell lung cancer (NSCLC), chronic lymphocytic leukemia (CLL), and other B-cell malignancies by covalently inhibiting kinase targets [10]. Numerous CKIs targeting 69 kinases have been designed and provided improved binding affinity to date. Meanwhile, the distribution of non-conserved nucleophilic amino acids (such as cysteine) across the whole kinome increase specificity and hence selectivity of kinase inhibitors [7,21,23,30,33,34]. In short, CKIs enrich selectivity over the whole kinome. Importantly there are more than 200 kinases over the kinome with available cysteines and other nucleophiles [33], which suggests there is plenty of room left for continued efforts to generate new covalent drugs [12,57]. Here, we reviewed CKI status, highlighting the privileged warheads that can be further used in the design of CKIs. 

The warhead moiety (electrophile) is the vital fragment of the CKI with the potential reactivity to the desired amino acids (nucleophile). Although establishing electrophiles has made great progress since 2011 [41,58,59,60], fast, precise prioritization of different nucleophiles to provide covalent linkage remains challenging. Difficulties arise when the active amino acid shows little reactivity, is difficult to access, or has a different local environment. Thus, more tailored novel warheads to tackle such targets are warranted.

We know cysteine is the most popular nucleophile when developing CKIs. However, other nucleophiles, such as lysine and tyrosine, can be utilized to react with the warheads in forming covalent interactions [61]. Warheads **25**–**28**, have been shown to target nucleophiles beyond cysteine. In particular, the catalytic lysine at the Catalytic_lys position was not only applied to design CKIs with the warheads **19**, **25**, **26**, and **27** but also to design the reversible CKIs with warhead **28** [62]. Because this catalytic lysine exists in every kinase, utilizing the lysine to develop CKIs may be advantageous [63]. It is worth noting that serine, threonine and tyrosine have long been used as covalent modification sites in various enzymes, such as hydrolases and transferases. Compared to lysine and cysteine, the alcohol side chains of serine, threonine and tyrosine have a modest nucleophilicity. During enzymatic catalysis, such as in a hydrolase, a catalytic triad within the catalytic site typically increases the alcohol nucleophilicity to easily react with the electrophile [64]. However, in CKIs, the serine, threonine, or tyrosine residues do not have a catalytic function [65]. Thus, a more reactive electrophilic group is expected to be required for reactions, especially with serine, threonine or tyrosine when compared to lysine or cysteine [66]. In this context, warhead **25** has gained much attention since it can target such hydroxy-containing amino acids despite having only modest nucleophilicity [42].

During the development process of CKIs, researchers are always concerned about the toxicity of CKIs because the covalent interaction will induce permeant modifications of the protein off-targets, if off-targets occur, and may cause haptenization [39]. A solution is to develop reversible CKIs, which not only retain the covalent interactions but also present manageable resident time. Reversible CKIs have drawn attention and target at least 10 kinases [40]. Moreover, two reversible CKIs, Rilzabrutinib (formerly PRN1008) and Roblitinib (known as FGF401), have been evaluated in clinical trials [56]. Given the potential toxicity of CKIs, it is desirable to design potentially less toxic reversible CKIs, and by extension, develop more warheads with covalent reversibility. The privileged warheads described here provide a valuable starting point to explore new nucleophiles and develop reversible warheads.

## Data Availability

The data presented in this study are available in the Supplementary Material Table S1.

## Supplementary Materials

The following supporting information can be downloaded at: https://www.mdpi.com/article/10.3390/ph15111322/s1, Table S1(xlsx), listing all of the released CKIs, with more detailed information included.
